# Magnetic Resonance Imaging Findings of Bilateral Cystic Neuroblastoma: Case Report of a Very Rare Entity

**DOI:** 10.7759/cureus.7073

**Published:** 2020-02-22

**Authors:** Esra Özgül

**Affiliations:** 1 Radiology, Afyonkarahisar Health Science University, Afyonkarahisar, TUR

**Keywords:** neuroblastoma, cystic, bilateral, rare, magnetic resonance

## Abstract

Neuroblastoma (NB) is the most common solid tumor seen in children under two years old. It has both solid and cystic forms. It generally involves the adrenal gland unilaterally. Only 10% of the NBs are as seen bilateral. Both bilateral and cystic forms of NB are a very rare entity. Magnetic resonance imaging (MRI) is a suitable imaging modality for evaluating lesions radiologically to avoid ionizing radiation exposure in children. Herein, we present the MRI findings of a bilateral cystic NB case.

## Introduction

Neuroblastoma (NB) is the most common extracranial tumor in infancy and childhood. It can arise anywhere in the sympathetic nervous system, including the adrenal glands [1]. It is commonly diagnosed in the infantile period and 25% of cases arise from the adrenal glands [2-3]. It presents in two forms: solid (56%) and cystic (44%). Less than 10% of NB tumors involve bilateral adrenal glands [1, 4]. Bilateral cystic NB (CNB) is a very rare entity that has large cystic adrenal lesions. Only a few cases of bilateral CNB have been described in the literature [1-7]. 

## Case presentation

A one-year-old girl was admitted to our hospital with abdominal distention of two days and abdominal discomfort of 10 days' duration. She was born at term by Caesarean section and her antenatal history was insignificant. Routine laboratory findings were within normal limits, except for elevated urine vanillyl mandelic acid (VMA) levels (157 mg/L).

On physical examination, a right abdominal mass was palpated. Sonography of the abdomen revealed bilateral adrenal anechoic cystic lesions with thick irregular walls. To avoid the ionizing radiation of computed tomography (CT), intravenous (IV) contrast-enhanced magnetic resonance imaging (MRI) was performed for further evaluation of the masses.

MRI findings

An MRI was performed with a 1.5-T MAGNETOM Aera unit with a body coil (Siemens, Erlangen, Germany). The contrast medium (gadoterate meglumine, 0.2 mL/kg (0.1 mmol/kg)) was infused through a 24-gauge venous catheter by using a power injector (Spectris) (Medrad® Inc., Warrendale, PA). Axial T1, coronal T1, coronal T2 true fast imaging with steady-state free precession (TRUFI), axial two-dimension time of flight (TOF), coronal three-dimension TOF, and precontrast and post-contrast fat-saturated axial and coronal T1-weighted images were taken with 3 mm section thickness.

**Figure 1:**
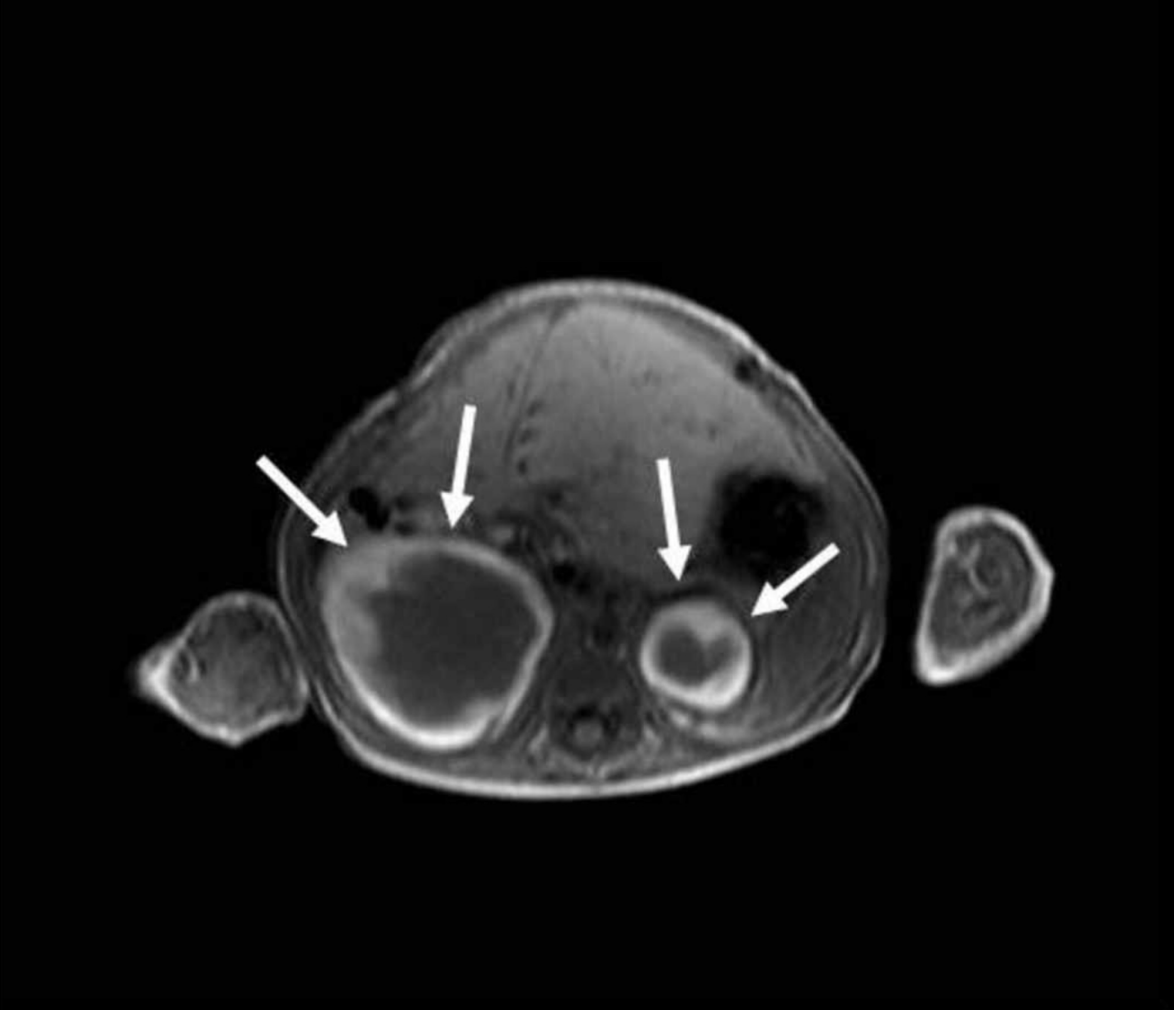
Bilateral adrenal cystic masses with hyperintense hemorrhagic changes are seen on axial T1-weighted image (arrows)

**Figure 2:**
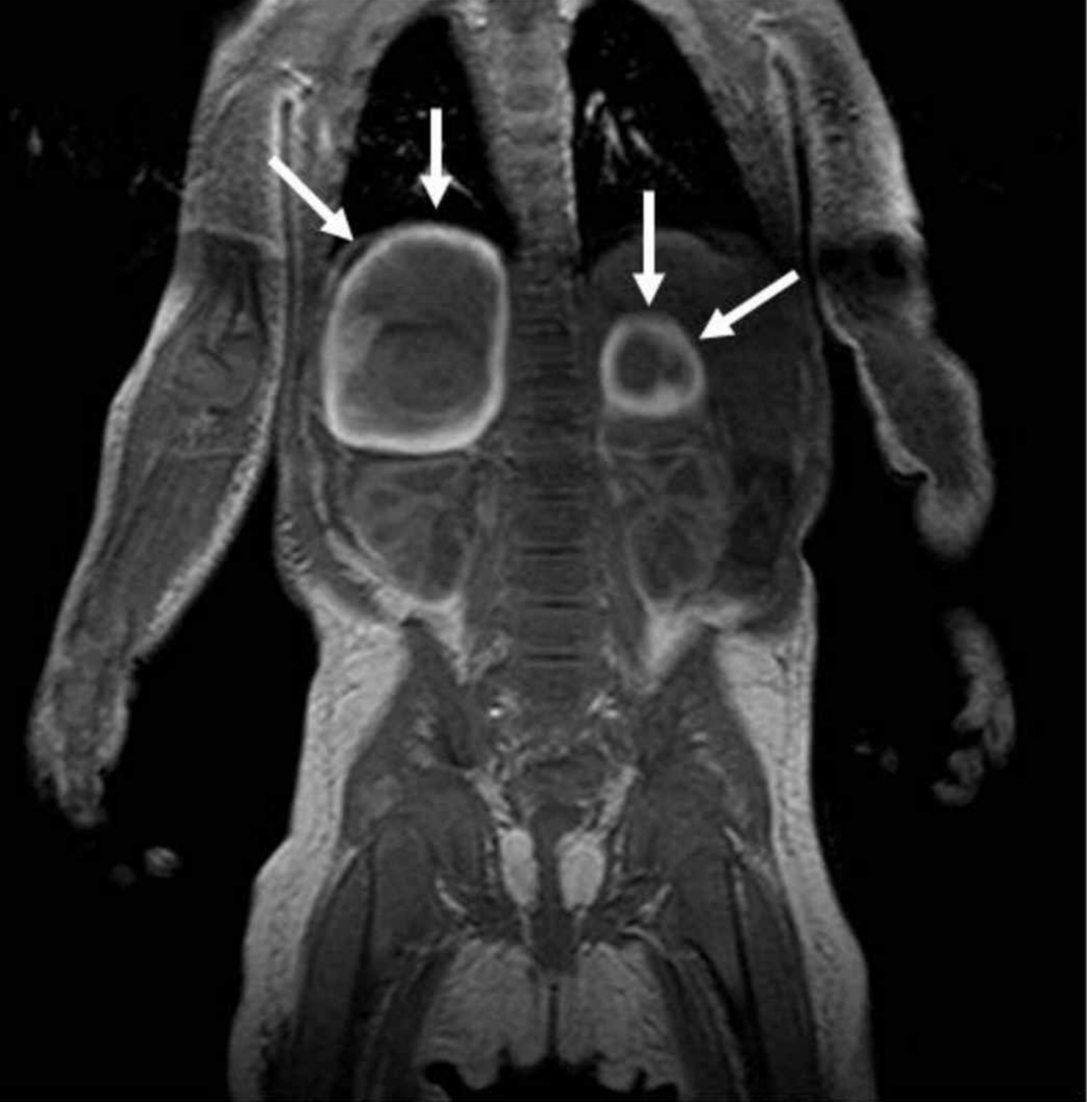
Bilateral adrenal cystic masses with hyperintense hemorrhagic changes are seen on coronal T1-weighted image (arrows)

**Figure 3:**
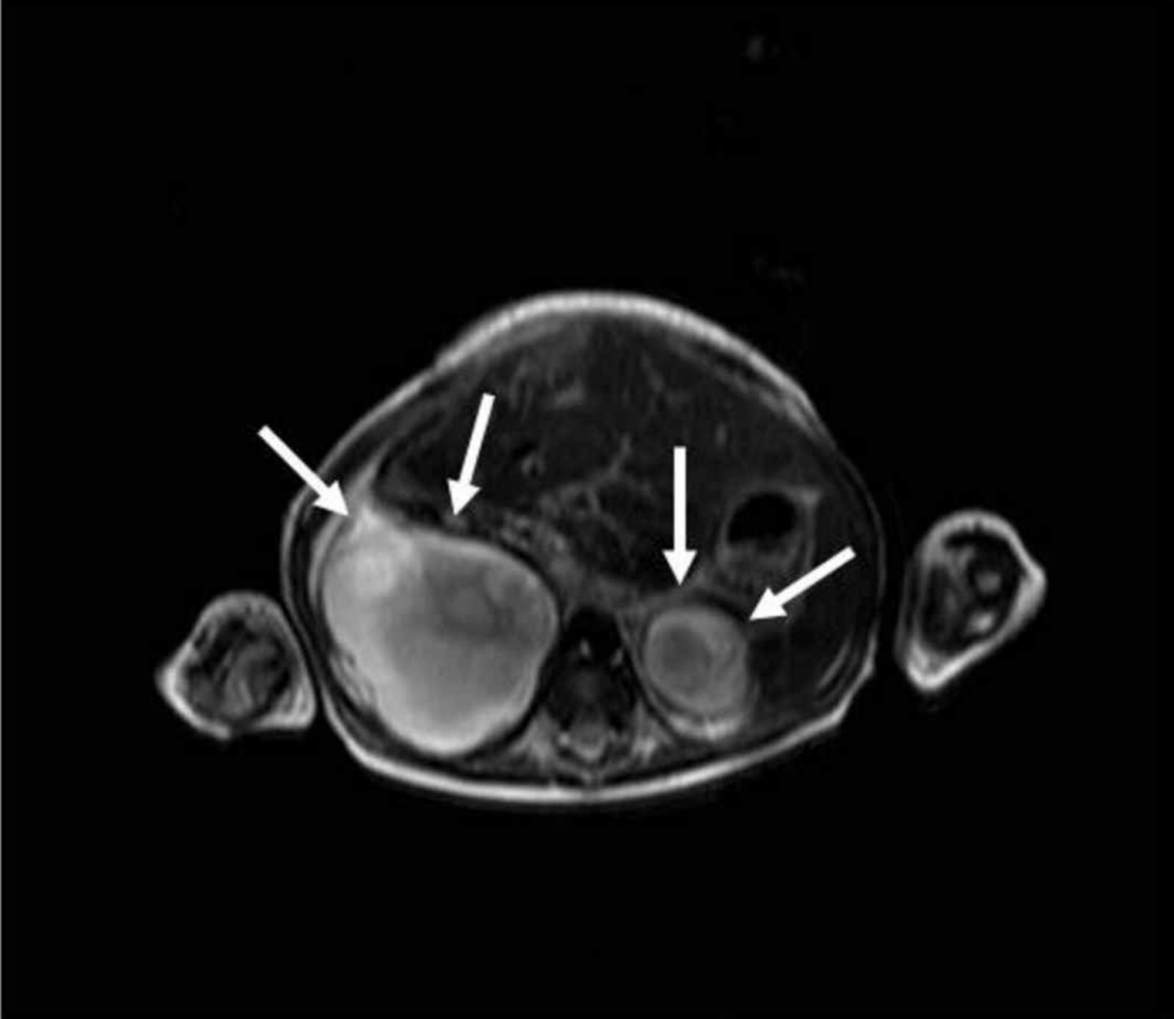
Bilateral adrenal cystic masses with hyperintense hemorrhagic changes are seen on axial T2-weighted image (arrows)

**Figure 4:**
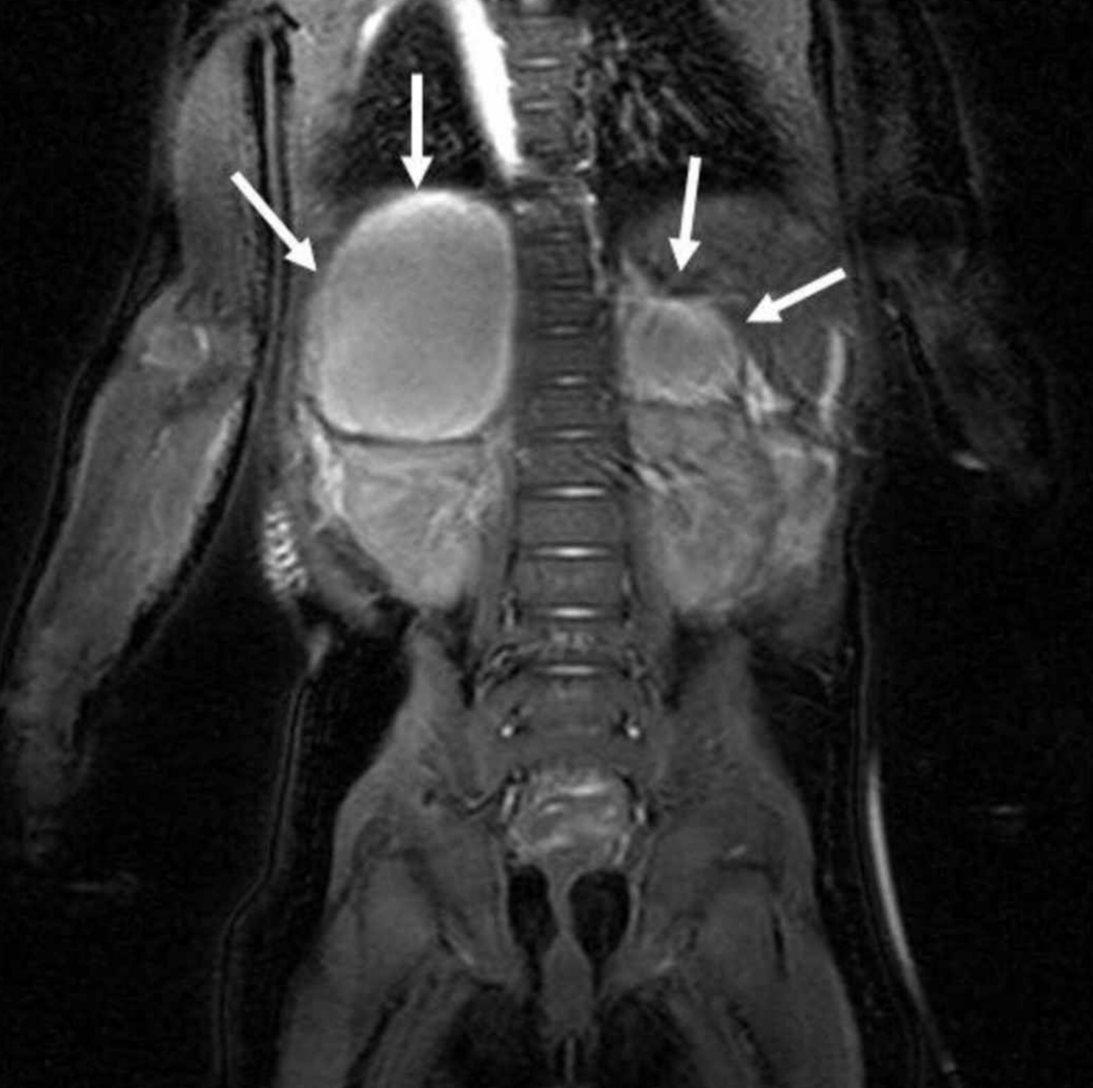
Bilateral adrenal cystic masses with hyperintense hemorrhagic changes are seen on coronal T2 TRUFI-weighted image (arrows) TRUFI: true fast imaging with steady-state-free precession

**Figure 5:**
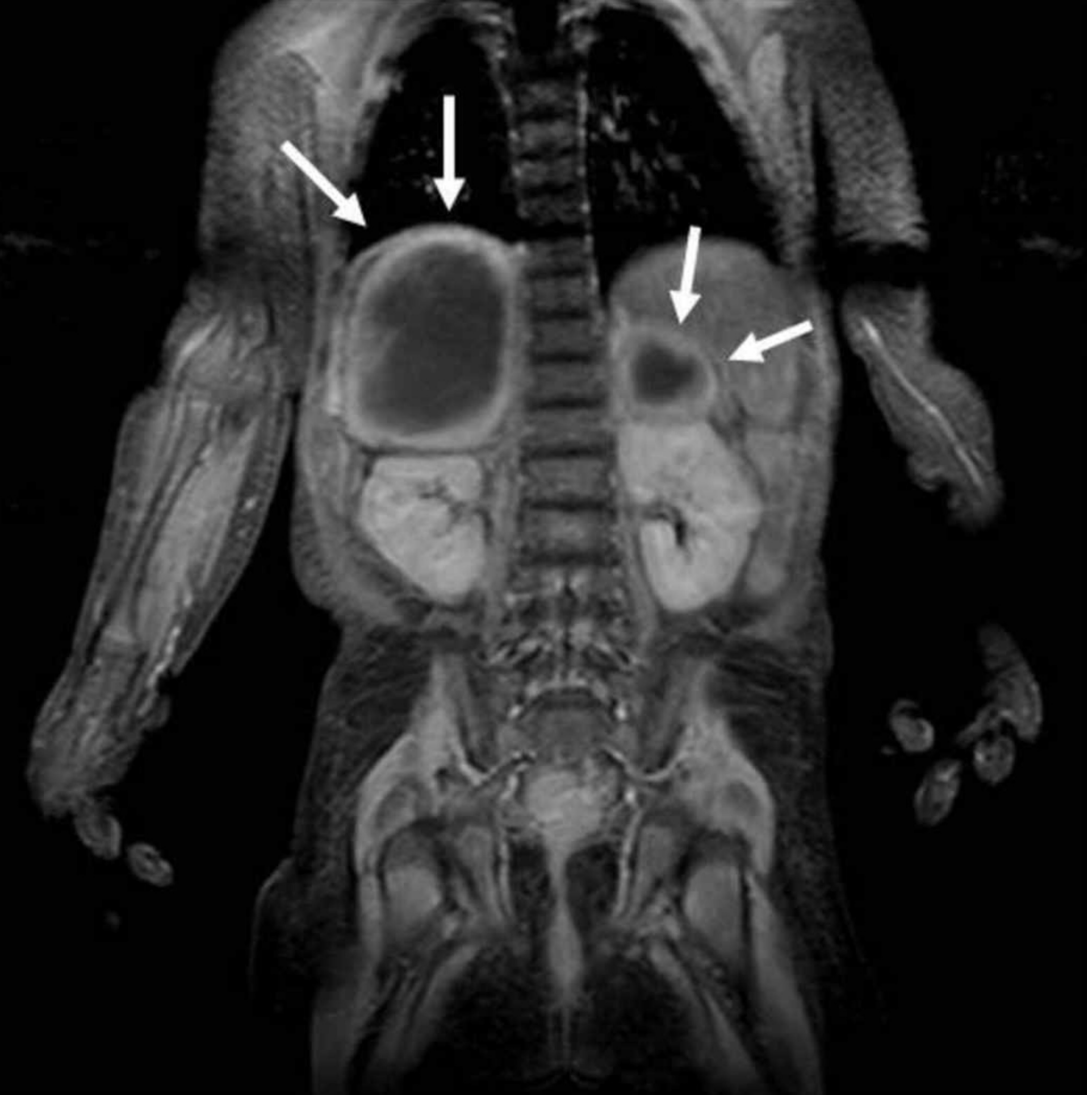
The right adrenal lesion was pushing the right kidney inferiorly. Both lesions had enhancing, thick, irregular walls without solid components on coronal post-contrast fat-saturated T1-weighted image (arrows)

**Figure 6:**
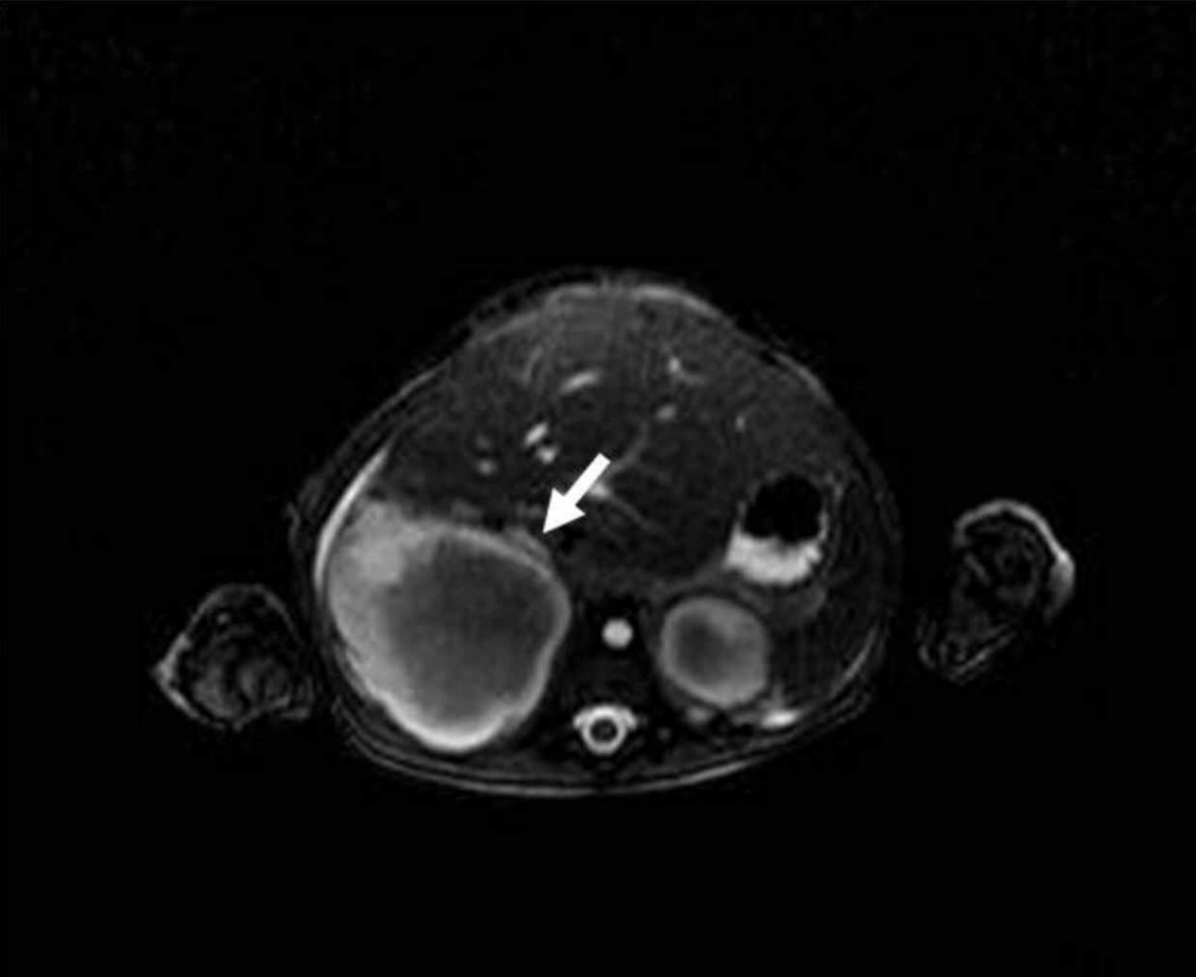
Axial two-dimensional time of flight (TOF) image shows narrowing and compression of the inferior vena cava (short arrow)

**Figure 7:**
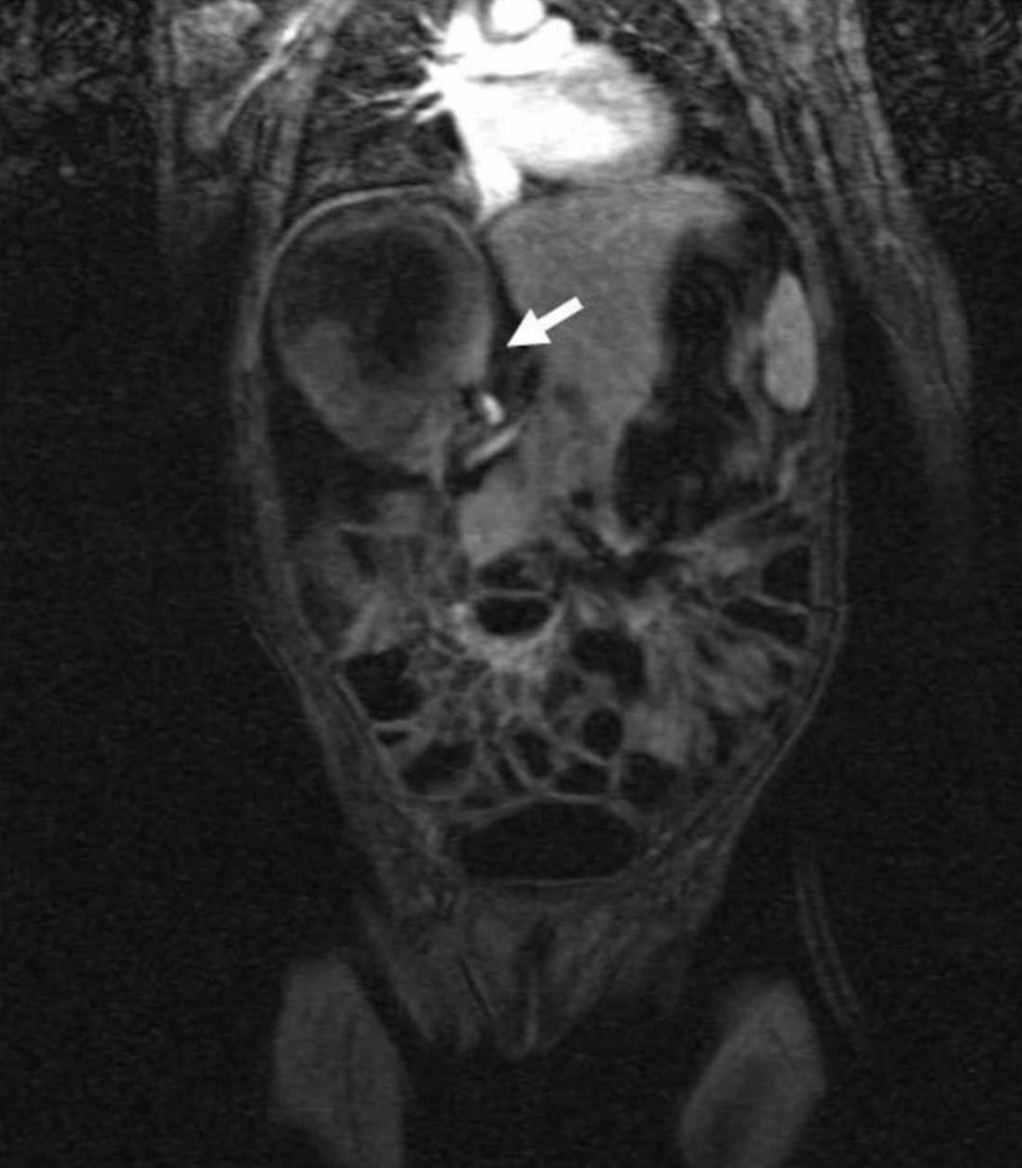
Coronal three-dimensional time of flight (TOF) magnetic resonance (MR) image shows narrowing and compression of the inferior vena cava (short arrow)

The MRI demonstrated a 6.5 x 5 x 5 cm right adrenal cystic mass and a 4 x 3.5 x 3 cm left adrenal cystic mass with hyperintense hemorrhagic changes on T1 and T2-weighted images. The right adrenal lesion was pushing the right kidney inferiorly. Both lesions had enhancing, thick, irregular walls. There were no solid components. The inferior vena cava was narrowed and compressed by the right adrenal mass. According to the MRI findings, the preliminary diagnosis was bilateral hemorrhagic CNB (Figures 1-7).

Ultrasound-guided fine-needle aspiration cytology (FNAC) was performed, and the diagnosis of NB with hemorrhagic changes was made pathologically. There was no evidence of lymph node or liver metastasis. The lesions were excised surgically. The patient was given chemotherapy after surgery and has been in remission for nine months.

## Discussion

NB is the most common malignant solid tumor of childhood and infancy. Nearly 50% of patients are under two years of age. The majority of patients reported in the literature are newborns [6]. The tumor may be solid (56%) or cystic (44%) [5]. Most of the patients reported had unilateral solid lesions. Only 10% of NBs have been documented as bilateral and bilateral involvement of the adrenal glands with cystic lesions is very rare [4].

Laboratory findings are generally insignificant. Vanillylmandelic acid (VMA) and homovanillic acid (HVA) levels are generally normal in CNBs. Ultrasound is the first step imaging modality for diagnosis. IV contrast-enhanced CT or MRI is performed for further diagnosis. However, because NB is a childhood tumor, an MRI should be performed instead of a CT to avoid ionizing radiation. Radiologic diagnoses were done by using a CT in most of the past studies, but there are a few reports in the literature about MRI findings of this entity [1-7]. MRI is the most suitable radiologic diagnostic method with no ionizing radiation. In this case report, we present the MRI findings of a bilateral cystic NB case. 

On MRI, the content of adrenal masses (solid or cystic), contrast enhancement pattern, presence of hemorrhage in the lesions, and metastasis of the masses can be easily detected.

Cystic forms have a more benign clinical course than solid forms [3]. Liver, bone, or lymph node metastasis can be seen in some cases [1-2, 7]. The differential diagnosis of CNBs are adrenal hemorrhage, dilated upper pole renal calyces, and extralobar sequestration [4].

A Tru-Cut® biopsy (Merit Medical, Jordan, UT) can be impossible in CNB if it has thin walls. FNAC or incision biopsy can be performed for pathologic diagnosis. Treatment generally consists of surgical resection with or without chemotherapy protocol.

## Conclusions

NB is the most common solid tumor seen in children under two years old. It has both solid and cystic forms and generally involves the adrenal gland unilaterally. The finding of both a bilateral and cystic form of NB, as was documented in this case report, is a very rare entity. MRI is the most suitable imaging modality for evaluating lesions radiologically to avoid ionizing radiation exposure in children. 
